# Guest edited collection: current friends and foes in gastrointestinal cancer

**DOI:** 10.1007/s12672-021-00428-3

**Published:** 2021-09-21

**Authors:** Giuseppe S. Sica

**Affiliations:** University of Rome “Tor Vergata”, Rome, Italy

Gastrointestinal cancers (GC) represent a heterogeneous complex array of disorders and diseases. They may be divided into rare inherited forms and more frequent sporadic forms. There is a critical interplay of genetic and environmental factors that foster the conversion of normal tissue to precursor, premalignant lesions, and eventually to frank malignancy. Moreover, the molecular mechanisms affecting the malignant progression are only partially known. While it is apparent that certain underlying genetic mechanisms, such as autophagy [1–3], apoptosis [4–6], metabolism [7–9], epigenetics [10, 11] and proteosiasis [12, 13], are better appreciated in a cell-type and tissue-type specific context, there are nevertheless overarching shared features between GC of different origin. GC are the second most common type of cancer, if men and women are considered together. There is general consensus that most GC develop over a long period of time, presenting with an attractive opportunity for intervention and prevention if diagnosed early. Since GC are diverse in aetiology, the clinical management is also different. However, epithelial-based GC represent the preponderant form of gastrointestinal malignancies and most of these cancers would benefit from surgical resections. The ‘Current Friends and Foes in Gastrointestinal Cancer Collection’ focus is on surgical treatment of the most common malignancies of the digestive tract, with an eye to cell-based pathways and hormone related cancer risk. Many thanks to all the authors for their contributions to this collection.

The role of radical surgery in the treatment of cancer is well established. With the tremendous forward strides in anaesthesia, fluid-balance, and antibiotic therapy, even older patients are tolerating more and more radical procedures, designed to eradicate both the local and distant effects of malignant disease. There are no medical contraindications to cancer surgery and also in emergencies it is possible to achieve oncologic clearance of neoplastic GC. In this respect, Di Carlo et al. [14] reviewed the surgical management of patients with perforated gastric cancer. Mortality rates ranged from 2 to 46%. However, patients able to receive an R0 resection showed better long-term survival compared with patients who had simple closure procedures. In emergency, curative RO resection should always be offered in patients without multiple adverse factors [15]. A surgical strategy using laparoscopic local repair as first step of surgery to resolve the peritonitis followed by a radical open or laparoscopic gastrectomy with lymphadenectomy could be considered. This paper highlights the importance of radical surgery also in emergency and results from this review should be taken into account for the treatment of acute abdomen due to GC.

Another important aspect of oncologic surgery is that of the correlation between volume and outcome, especially for tumours that require certain specific organization, up-to-date technology and experience with minimally invasive surgery. One such tumour is the cancer of the rectum, as discussed in the research by Siragusa et al [16], who found that estimated leak rate was significantly lower in higher volume unit and that mean operative time, need of conversion, postoperative use of blood transfusion and in-hospital stay were significantly reduced, increasing the number of operations performed. It is certainly an interesting finding considering that Hospital centralization effect is reported to lower complications and mortality for high risk and complex surgery operations, including colorectal surgery, but no linear relation between volume and outcome was demonstrated so far. Potentiation of existing low/medium volume surgical departments able to provide standard care for rectal cancer, by operational preparation and strategic planning, can yield improved perioperative results.

For what concern innovative cell-based research and bench to bedside projects, in this collection we included an interesting experiment from Beijing Friendship Hospital, Capital Medical University, China [17]: Dr. Wang and co-authors studied the possibility to sensitize colorectal cancer cells towards 5-fluorouracil (5-Fu), a commonly used chemotherapeutic agent employed in most protocols against GI cancers. The group coordinated by Dr. Li, used nephroblastoma-overexpressed protein (NOV) to overcome the problem of chemo resistance often seen during 5-Fu treatment of colorectal cancer (CRC). Nude mouse xenograft model was established to test the inhibitory effect of 5-FU on CRC cells in vivo. As often happen in this type of research [18, 19], tumour specimens and adjacent normal colorectal tissue were collected during surgery and RNA was extracted. The cell suspensions of CRC cells stably transfected with NOV amongst others, were collected and inoculated into the left lateral skin of nude mice to establish the nude mouse model. One week later, the nude mice were treated with 5-Fu and subsequently the body weight and tumour volume of the nude mice were monitored after inoculation for 8 weeks. The authors found that NOV improved the inhibitory effect of 5-Fu on CRC cells in vitro and improved the inhibitory effect of 5-Fu in tumour cell xenotransplantation nude mouse model. Clearly, drugs targeting NOV need to be explored and applied in further clinical trials to fully illuminate the actual clinical therapeutic effect but results such as this one provides an experimental basis for the potential use of NOV as a target for the promotion of 5-Fu treatment.

Along with clinical and experimental research studies, we have received and included in the GI cancer collection, two meta-analysis. The aim of the first systematic review and meta-analysis was to investigate the associations between endogenous concentrations of sex hormones and CRC risk. PubMed and Scopus were searched until June 2020 for prospective studies evaluating the association between pre-diagnostic plasma/serum concentrations of estradiol, testosterone and sex-hormone binding globulin (SHBG) and CRC risk [20]. Even though preclinical data suggest that endogenous sex steroid hormones may be implicated in colorectal cancer (CRC) development however, findings from this meta-analysis do not support presence of associations between pre-diagnostic concentrations of testosterone, estradiol and SHBG with incident CRC risk in men and post-menopausal women.

The second meta-analysis was on the correlation of histological subtypes of CRC with varied prognostic features [21]. This systematic review and meta-analysis aimed to compare clinicopathological characteristics, recurrence and overall survival between colorectal signet-ring cell (SC) and mucinous carcinoma (MC) to conventional adenocarcinoma (AC). Meta-analysis was performed using random-effect models and between-study heterogeneity was assessed. Based on the available evidence, SC and MC present and behave in a pattern distinct from AC. SC seems to be an aggressive type of CRC presenting in younger patients, in the right colon and at a more advanced stage. This in turn leads to a poorer stage-by-stage survival and a higher probability of local recurrence rates when compared to AC. MC seems to behave in a similar fashion to SC in terms of local recurrence and overall survival. These factors need to be taken into consideration when planning surgical and oncological management of such cancers.

The other four articles of this collection on GI cancers are based on clinical studies. The group from Portsmouth in UK, coordinated by J Khan, highlight the equivalent oncological outcomes of low rectal resection when compared to the most invasive amputation of the rectum and anus [22]. The so called abdominal perineal excision of rectum is an operation popularized by Sir E. Miles back in 1908 remained the gold standard for the treatment of cancers of the lower rectum for a century, but it certainly carries important implications due to the permanent stoma, affecting patient’s quality of life. Furthermore, the authors found a lower incidence of post-operative complication when performing a sphincter saving operation and suggest a tailored approach suited to the individual patients needs, supported by the multidisciplinary team.

Another important chapter in colon and rectal surgery is that of post-surgical complications. Ikeda et al. from Tokyo cancer centre, offered to the collection a prospective observational study aimed to clarify the incidence and independent risk factors of wound infection after laparoscopic surgery for primary colonic and rectal cancer [23]. Surgical site infection is of paramount importance for each and any surgical procedure and often represent a key performance indicator for quality assessment [24]. In this prospective report the outcomes of interest were the incidence and risk factors of wound infection. The authors found that laparoscopic rectal surgery has a higher risk of wound infection than colonic surgery and the key message is that laparoscopic rectal surgery involving abdominoperineal resection, patients with higher BMI, and chemoradiotherapy requires careful observation in wound care and countermeasures against wound infection.

A different type of cancer of the digestive tract is Anal Squamous Cell Carcinoma (ASCC), a rare cancer that has a rapidly increasing incidence in areas with highly developed economies. ASCC is strongly associated with HIV and there appears to be increasing numbers of younger male persons living with HIV diagnosed with ASCC. Brogden and co-authors, present their experience with managing patients with ASCC over the last two decades with emphasis on whether there exists a separate subpopulation of ASCC patients with HIV within the Trust and whether this affects clinical prognosis [25]. This is a large cohort of patients with a rare cancer, in a specialist centre for the management of HIV and ASCC. The data available permitted the review of a 20-year experience of treating ASCC in a demographic where HIV is highly prevalent. There is little previous data comparing the outcomes between HIV positive and HIV negative patient groups. Results from this cohort identified a patient subset of younger male PLWH developing ASCC. Patients known to have a previous diagnosis of AIN were more likely to have earlier disease. There was no statistical difference in staging and disease-free survival and HIV status. However, younger male persons living with HIV were more likely to recur and when this occurred, it took longer to develop recurrent disease.

Gallbladder carcinoma is often found incidentally on histopathologic examination after cholecystectomy: this is referred as incidental gallbladder carcinoma (IGC). Routine vs selective histopathological assessment of gallbladders is under debate and the study from Dr. Manzelli’s team, evaluates the role of regular specimens’ examination, based on a single-centre analysis of incidence, clinical and histopathological aspects of IGC [26]. They examined the pathology reports of almost 6000 gallbladder specimens, finding the incidence of IGC as low as 0.1%. The authors argue that a high volume of cholecystectomies might prevent GB cancer by interrupting the progression of chronic inflammation and dysplasia towards malignant transformation, thus leading to a reduced incidence. However, the study was not powered for such evaluation and further research is needed to corroborate or confute such a hypothesis. Given the relative low incidence of IGC, there is ongoing debate as to whether assessment of all the GB specimens should be done on a regular basis [27]. Arguments in favour of a routine approach include the lack of potential oversight of cases, accurate disease staging and medico-legal implications in case of disputes or diagnostic uncertainty. The authors suggest a more selective approach that entails the examination of the specimen only if the GB looks abnormal pre- or intraoperatively (i.e., in the presence of a thick, fibrosed wall, local dense adhesions). Such a strategy would bring a reduction for national health systems with minimum risks.

In conclusion, the ‘Current Friends and Foes in Gastrointestinal Cancer Collection’ gives a broad overview of different, current and future, perspectives on tumours of the digestive system. It comprises studies investigating different gastrointestinal malignancies and it includes up-to-data basic scientific cell experiments, cohort data and clinical researches aimed to unveil underlying molecular mechanisms as well as to ameliorate clinical applications, mainly in the field of surgery.
